# Dirac metallic FeB_2_-induced low Schottky barrier and electrically tunable Schottky contact in FeB_2_/MoS_2_ van der Waals heterostructure

**DOI:** 10.1039/d6na00090h

**Published:** 2026-05-06

**Authors:** Tran T. Nhan, Nguyen Q. Cuong, Chuong V. Nguyen, Huynh V. Phuc

**Affiliations:** a Faculty of Fundamental Sciences, Hanoi University of Industry 298 Cau Dien Hanoi 100000 Vietnam tran.nhan@haui.edu.vn; b Institute of Research and Development, Duy Tan University Da Nang 550000 Vietnam nguyenquangcuong3@duytan.edu.vn; c School of Engineering & Technology, Duy Tan University Da Nang 550000 Vietnam; d Department of Materials Science and Engineering, Le Quy Don Technical University Hanoi 100000 Vietnam; e Division of Physics, School of Education, Dong Thap University Cao Lanh 870000 Vietnam hvphuc@dthu.edu.vn

## Abstract

In this work, we employ first-principles density functional theory (DFT) calculations to systematically investigate the interfacial electronic properties and contact behavior of a Dirac-metallic Dirac-FeB_2_/MoS_2_ van der Waals (vdW) heterostructure. The Dirac-FeB_2_/MoS_2_ system is found to be energetically, mechanically, thermally, and dynamically stable, indicating its potential experimental feasibility. Notably, the heterostructure forms an n-type Schottky contact with an ultralow electron barrier height of 0.125 eV and a low tunneling resistance of 1.82 × 10^−9^ Ω cm^2^. This superior contact performance is attributed to the delocalized Dirac electrons and the weak Fermi-level pinning at the interface, providing key insight into the role of Dirac metals in contact engineering. Furthermore, the Schottky barrier can be effectively tuned by an external electric field, enabling a reversible transition from Schottky to ohmic contact. These findings highlight the promise of Dirac metallic FeB_2_ as an efficient electrode material and offer practical guidance for the design of high-performance 2D nanoelectronic and optoelectronic devices with reduced contact resistance.

## Abstract 2

In this work, we employ first-principles density functional theory (DFT) calculations to systematically investigate the interfacial electronic properties and contact behavior of a Dirac-metallic Dirac-FeB_2_/MoS_2_ van der Waals (vdW) heterostructure.

## Graphical abstract

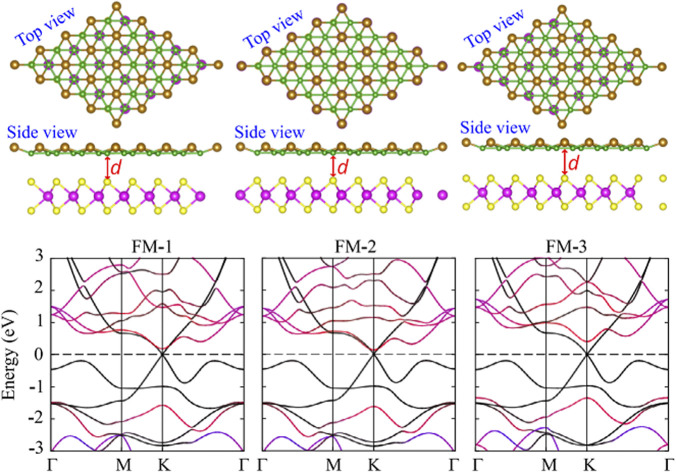

## 1 Introduction

In recent decades, two-dimensional (2D) materials have become a cornerstone of condensed matter physics and materials science owing to their unique and versatile properties.^1–4^ The confinement of charge carriers within atomically thin layers gives rise to electronic, optical, and mechanical characteristics that markedly differ from their bulk counterparts. Prominent examples include graphene,^5^ phosphorene,^6^ transition metal dichalcogenides (TMDs),^7^ and MXenes,^8^ which have all attracted intensive attention. These materials are not only scientifically intriguing but also hold transformative potential for applications ranging from flexible electronics and photodetectors to quantum computing and energy harvesting. Despite their promise, the functionalities of individual 2D materials remain limited, often constraining their application in advanced device architectures. For example, the absence of a sizable band gap in graphene significantly hampers its use in logic devices requiring a high on/off current ratio.^9^ Similarly, although phosphorene exhibits a tunable direct band gap and high carrier mobility,^10^ its intrinsic instability under ambient conditions severely limits practical applications.^11^ In the case of MoS_2_, while the presence of a sizable band gap makes it attractive for field-effect transistors and optoelectronic devices,^12^ its relatively low carrier mobility remains a critical bottleneck for high-performance applications.^13^

To overcome these limitations and unlock new functionalities, the creation of van der Waals (vdW) heterostructures by stacking^14,15^ or integrating^16,17^ different 2D materials has become a major research focus. Vertical heterostructures, formed by stacking individual layers, are particularly advantageous for investigating interfacial charge transfer, band alignment, and Schottky barrier formation across atomically sharp interfaces. In contrast, lateral heterostructures, where different materials are seamlessly joined within the same plane, are more suitable for studying in-plane transport and junction properties but typically require more complex structural modeling and fabrication considerations. By suitable selecting and combining different layers, researchers can tailor the physical properties and induce interfacial phenomena that are absent in the individual constituents. With continuous advances in fabrication techniques and increasingly accurate computational models, the rational design of vdW heterostructures is expected to enable multifunctional devices. Among various configurations, metal–semiconductor vdW heterostructures are of particular interest due to their crucial role in tuning contact resistance, band alignment, and carrier injection in next-generation electronic and optoelectronic devices.^18,19^

Recently, a novel boron-based 2D Dirac FeB_2_ material has been reported, in which planar hexacoordinate iron (Fe) atoms are embedded within a boron honeycomb lattice structure.^20^ Monolayer FeB_2_ hosts a Dirac cone near the Fermi level, giving rise to massless Dirac fermions with a remarkable Fermi velocity of 6.54 × 10^5^ m s^−1^, thereby enabling ultrafast charge transport.^20^ In addition, monolayer FeB_2_ exhibits metallic behavior with excellent electrical conductivity and ultrahigh carrier mobility on the order of 10^5^ cm^2^ V^−1^ s^−1^, which is comparable to that of graphene.^21^ Notably, nanosheet FeB_2_ can also be synthesized in recent experiment^22^ and it has been predicted to serve as a promising electrode material for metal-ion batteries.^23^ All these findings suggest that FeB_2_ is not only of fundamental interest as a novel 2D Dirac metal but also holds great promise as a metallic building block for integration into 2D metal–semiconductor heterostructures.

Currently, the integration of 2D metals with TMDs semiconductors has attracted considerable attention owing to the unique electronic and optical properties of TMDs as well as their relatively stable and scalable synthesis.^24–26^ Such metal–semiconductor heterostructures provide a versatile platform for exploring interfacial physics and developing high-performance electronic and optoelectronic devices. Among the family of 2D TMD semiconductors, MoS_2_ is the most widely studied due to its sizable band gap^27^ and outstanding mechanical behavior.^28^ Recently, heterostructures combining two-dimensional (2D) metals with MoS_2_ semiconductors have been successfully fabricated and investigated.^29,30^ These studies demonstrated that MoS_2_ serves as an efficient channel material for integration with 2D metallic layers. Therefore, in this work, we design the integration of metal–semiconductor heterostructure between 2D metallic FeB_2_ and 2D semiconductor MoS_2_ using first-principles calculations.

## 2 Computational details

First-principles calculations based on density functional theory (DFT) were performed using the PWscf package within the Quantum ESPRESSO distribution.^31,32^ The exchange–correlation interaction was treated within the generalized gradient approximation (GGA) using the Perdew–Burke–Ernzerhof (PBE) functional.^33^ The electron–ion interactions were described by projector augmented wave (PAW) pseudopotentials. A plane-wave basis set with a kinetic energy cutoff of 510 eV was employed to ensure convergence of total energies. Brillouin zone integrations were carried out using a Monkhorst–Pack *k*-point mesh of 15 × 15 × 1. A vacuum spacing of 30 Å was introduced along the out-of-plane direction to avoid spurious interactions between periodic images. All structures were fully relaxed until the residual force on each atom was less than 0.01 eV Å^−1^, and the total energy convergence criterion was set to 10^−8^ eV. Spin–orbit coupling (SOC) effects were also included in the calculations to account for relativistic interactions, which are known to play a crucial role in transition metals. To investigate vibrational properties and dynamical stability, phonon spectra were calculated within density functional perturbation theory (DFPT).^34,35^ Phonon dispersions were obtained using a 3 × 3 × 1 *q*-point mesh.

## 3 Results and discussion

**Fig. 1:**
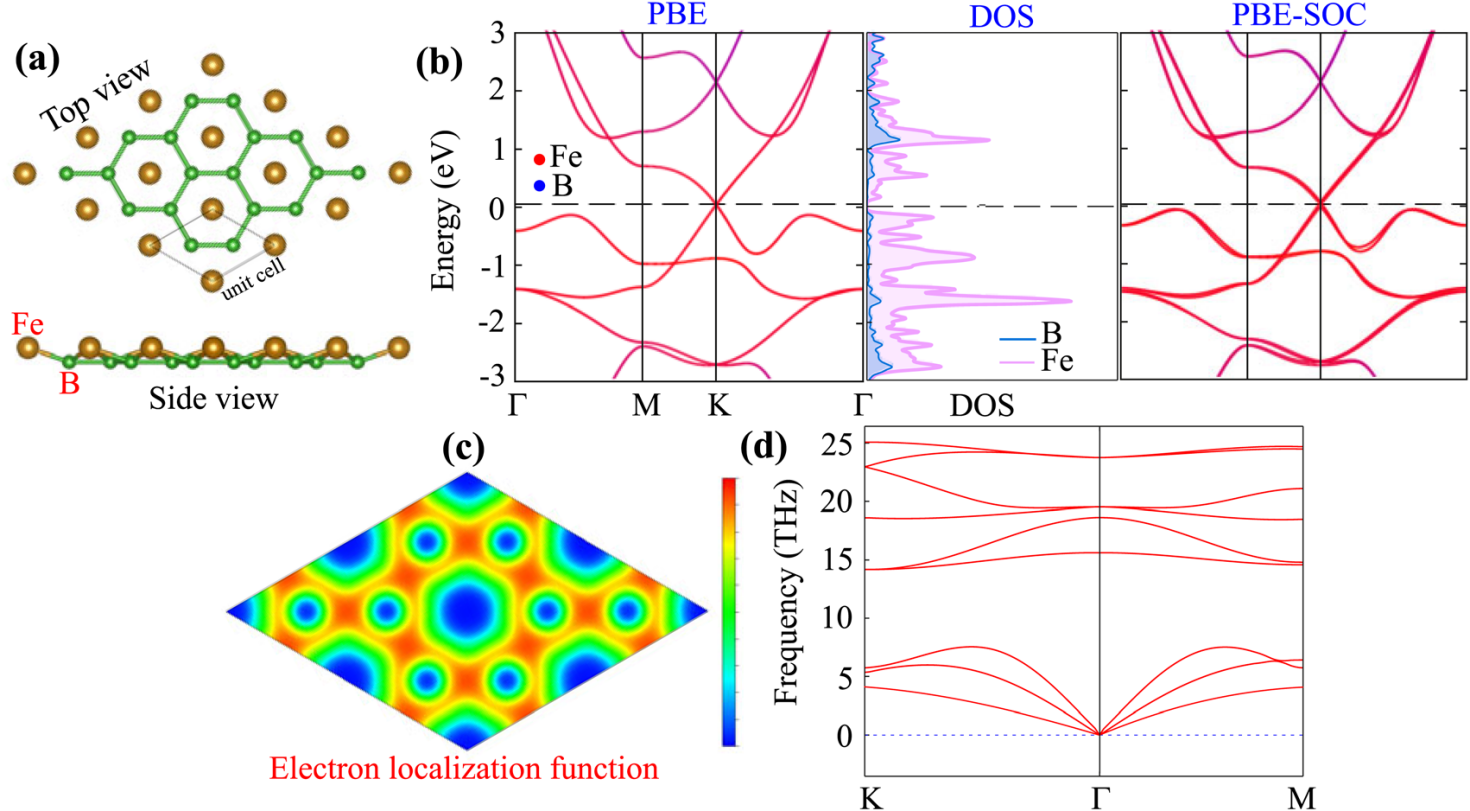
(a) Optimized geometry, (b) projected band structures without and within the presence of SOC effect and the projected density of states, (c) electron localization function and (d) phonon spectrum of FeB_2_ monolayer.

We first investigate the structural, electronic, and dynamical stability of monolayer FeB_2_ as a prerequisite for constructing the FeB_2_/MoS_2_ heterostructure. Upon full structural optimization, Fe atoms remain embedded within the boron honeycomb lattice, forming a planar hexacoordinate geometry in good agreement with previous reports.^20,22^ The optimized lattice constant is determined to be 3.18 Å, consistent with earlier theoretical predictions.^20,22^ The FeB_2_ monolayer crystallizes in the *P*6/3*m* space group. Its unit cell consists of one Fe atom embedded within a two-dimensional hexagonal boron lattice, as illustrated in Fig. 1(a). The vertical distance between the Fe atom and the boron hexagonal plane is calculated to be 0.63 Å, which is in excellent agreement with previous theoretical predictions,^20^ thereby validating the reliability of our computational approach. The electronic band structures of FeB_2_ monolayer without and within the presence of SOC effect are illustrated in Fig. 1(b). Both PBE and PBE-SOC reveal that FeB_2_ monolayer exhibits a metallic behavior with a Dirac cone located near Fermi level at the *K* point. When SOC effect is included, a band splitting emerges near the Dirac point, but the metallic nature of the system remains intact. The projected density of states (PDOS) of FeB_2_ monolayer indicate that its Dirac states near the Fermi level is mainly contributed by the Fe-d orbital states. To further understand the physical origin of the FeB_2_ monolayer, we analyzed its electron localization function (ELF), as shown in Fig. 1(c). The results reveal that the FeB_2_ monolayer is characterized by strong covalent bonding within the hexagonal boron plane, while the Fe atom exhibits delocalized electron distribution with weak interaction with the surrounding boron atoms. Furthermore, to further evaluate the dynamical stability of FeB_2_, phonon dispersion calculations were carried out within the framework of DFPT, as displayed in Fig. 1(d). The absence of imaginary frequencies across the entire Brillouin zone confirms that monolayer FeB_2_ is dynamically stable and, in principle, experimentally realizable.

**Fig. 2:**
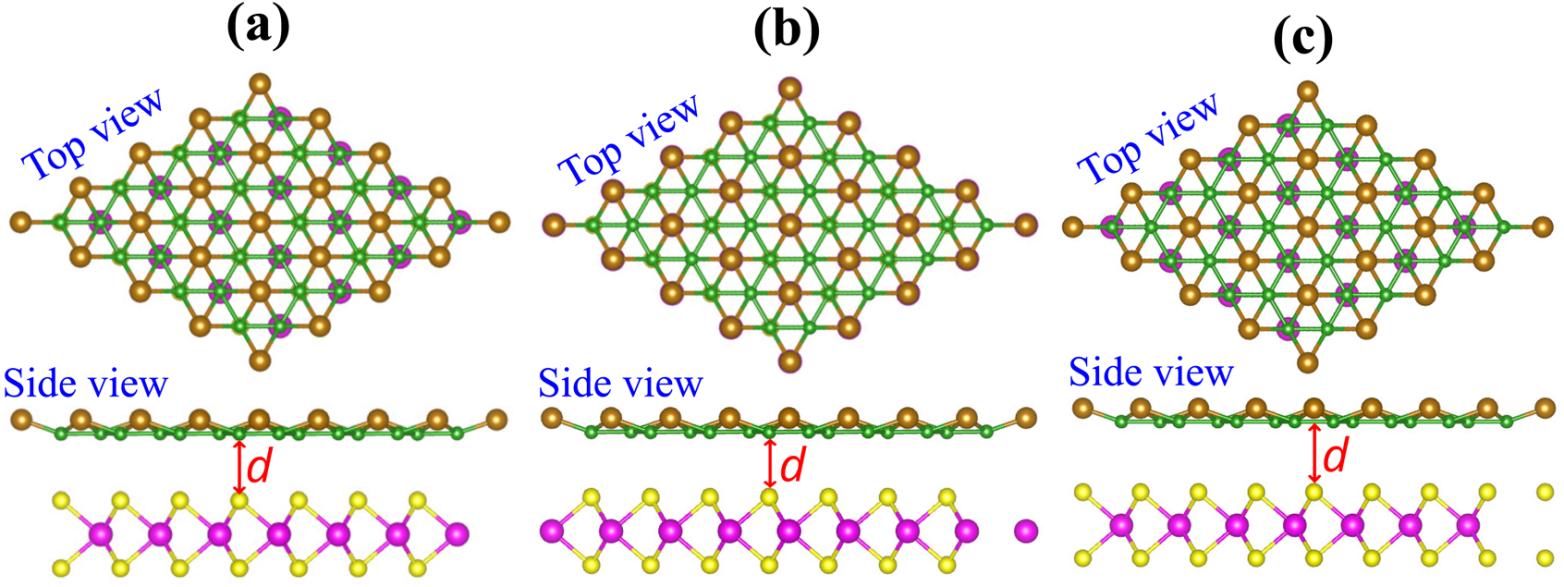
Optimized atomic structures of Dirac states metal FeB_2_/MoS_2_ heterostructures for different stacking configurations of (a) FM-1, (b) FM-2 and (c) FM-3.

We further design a metal–semiconductor FeB_2_/MoS_2_ heterostructure by vertically stacking the Dirac metallic FeB_2_ monolayer on top of semiconducting MoS_2_. Three possible stacking configurations of the FeB_2_/MoS_2_ heterostructure are illustrated in Fig. 2. In these models, the Fe atoms are positioned at different high-symmetry sites relative to the Mo and S atoms of the MoS_2_ layer, namely FM-1, FM-2 and FM-3. After full structural relaxation, the interlayer distances (*d*) between the topmost S layer of MoS_2_ and the bottom B layer of FeB_2_ in the Dirac-FeB_2_/MoS_2_ heterostructure are found to be 3.06, 3.00, and 3.34 Å for the FM-1, FM-2, and FM-3 stacking configurations, respectively. It is evident that among these configurations, the FM-2 stacking exhibits the shortest interlayer spacing. Moreover, the obtained interlayer spacing for all configurations fall within the typical range of vdW interactions, confirming the weakly bonded nature of the Dirac-FeB_2_/MoS_2_ heterostruture. Furthermore, to quantitatively evaluate the interlayer coupling and relative stability, we calculated the binding energies (*E*_b_) of these stacking configurations using the expression:1
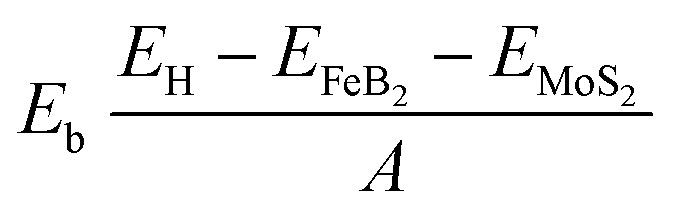
Here, *E*_H_, *E*_FeB_2__, and *E*_MoS_2__ represent the total energies of the Dirac-FeB_2_/MoS_2_ heterostructure, and the isolated FeB_2_ and MoS_2_ monolayers, respectively. The calculated binding energies of the Dirac-FeB_2_/MoS_2_ heterostructure for the FM-1, FM-2, and FM-3 stacking configurations are found to be −25.83, −27.66, and −21.41 meV Å^−2^, respectively. The negative values of the binding energies indicate that all the stacking configurations are energetically favorable and stable. Among them, the FM-2 configuration exhibits the lowest binding energy, confirming that it is the most energetically preferred stacking arrangement for the Dirac-FeB_2_/MoS_2_ heterostructure. In addition, these values are comparable to those obtained in other typical vdW heterostructures, such as PbN/CdO,^36^ NbSe_2_/MoS_2_,^37^ SiC/borophene,^38^ AlN/Zr_2_CO_2_ (ref. 39) and Ti_2_C/MoS_2_,^40^ confirming that the interaction between FeB_2_ and MoS_2_ layers is dominated by weak vdW forces rather than chemical bonding.

**Fig. 3:**
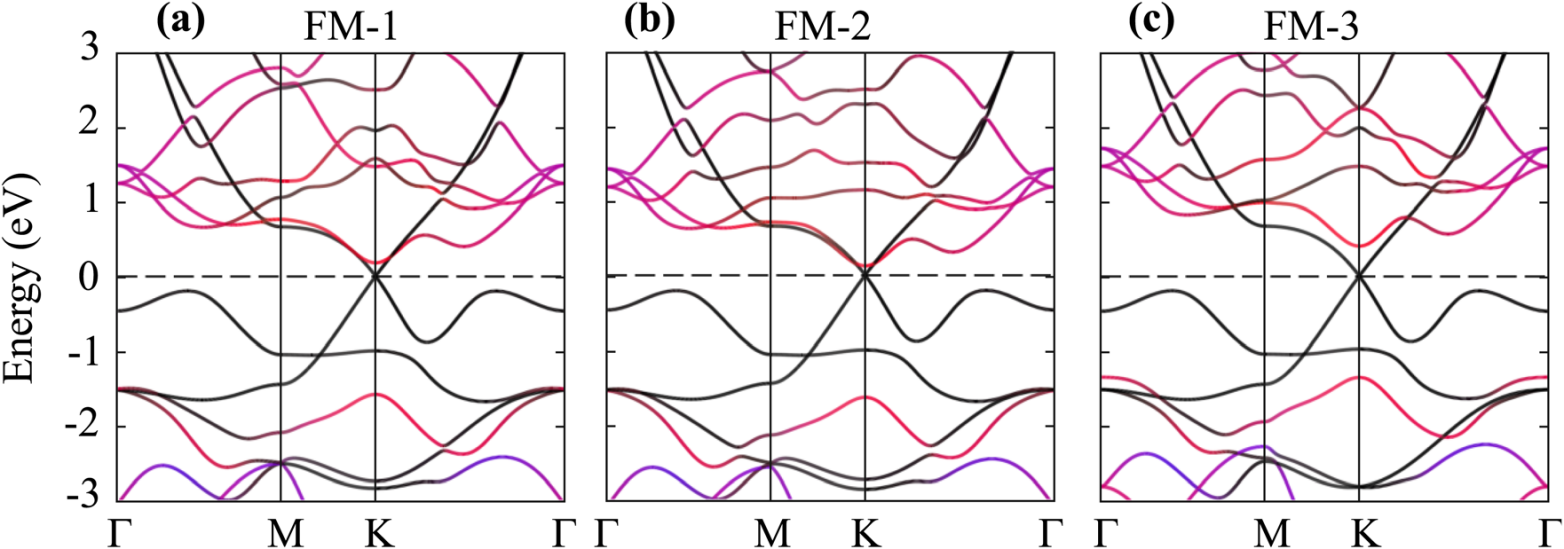
Projected band structures of the Dirac-FeB_2_/MoS_2_ heterostructure for (a) FM-1, (b) FM-2 and (c) FM-3 stacking configurations. The contributions of the Dirac-FeB_2_ and MoS_2_ layers are marked by black and red-blue lines, respectively. The Fermi level is set to be zero and marked by the dashed black line.

**Fig. 4:**
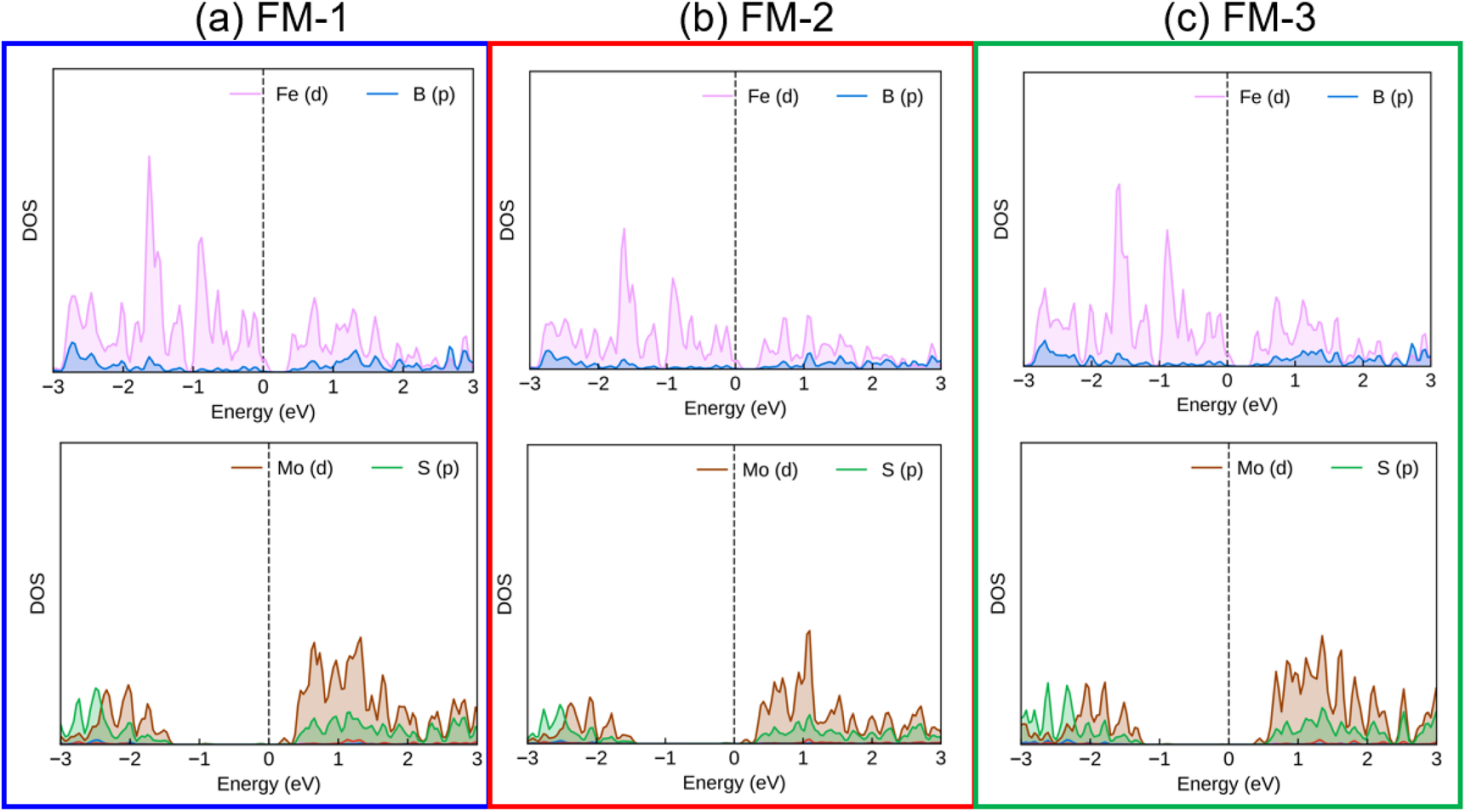
PDOS of all atoms in the Dirac-FeB_2_/MoS_2_ heterostructure for (a) FM-1, (b) FM-2 and (c) FM-3 stacking configurations.

We further investigate the electronic properties of the Dirac-FeB_2_/MoS_2_ heterostructures under different stacking configurations by analyzing their projected band structures and atom-resolved density of states (DOS), as illustrated in Fig. 3 and 4. It is evident that all stacking configurations preserve the metallic character of the heterostructure, with a distinct Dirac cone located at the *K* point. The Dirac cone originates predominantly from the FeB_2_ layer, indicating that the intrinsic Dirac feature of FeB_2_ is well retained upon interfacing with the MoS_2_ monolayer. The similar preservation was also observed in other vdW metal–semiconductor heterostructures, such as graphene/GaN.^41^ Likewise, the semiconducting nature of the MoS_2_ layer remains almost unaffected. This preservation of the intrinsic electronic characteristics of both components can be attributed to the weak van der Waals interaction at the Dirac-FeB_2_/MoS_2_ interface, which prevents significant orbital hybridization between the two layers.

Interestingly, a metal–semiconductor junction is naturally formed at the Dirac-FeB_2_/MoS_2_ interface, which plays a vital role in tailoring the charge transport characteristics and designing next-generation nanoelectronic and optoelectronic devices. At the Dirac-FeB_2_/MoS_2_ interface, Schottky-type contacts with narrow barrier heights are formed in all stacking configurations. This behavior arises because the Fermi level of the metallic Dirac-FeB_2_ layer lies between the conduction and valence band edges of the semiconducting MoS_2_ layer. The Schottky barriers at the metal–semiconductor interface are defined as follows:

2 *Φ*^e^_SB_ = *E*_C_ − *E*_F_, *Φ*^h^_SB_ = *E*_F_ − *E*_V_‬‬‬‬‬‬‬‬‬‬‬‬‬‬‬‬‬‬‬‬‬‬‬‬‬‬‬‬‬‬‬‬‬‬‬‬‬‬

where *Φ*^e^_SB_ and *Φ*^h^_SB_ represent the electron and hole Schottky barrier heights, respectively. Here, *E*_C_ and *E*_V_ denote the energies of the conduction band minimum (CBM) and valence band maximum (VBM) of the MoS_2_ layer, while *E*_F_ is the Fermi level of the Dirac-FeB_2_/MoS_2_ heterostructure. The calculated Schottky barriers *Φ*^e^_SB_ (*Φ*^h^_SB_) for the FM-1, FM-2, and FM-3 configurations are found to be 0.179 (1.679), 0.125 (1.665) and 0.404 (1.754) eV, respectively. It is obvious that the Schottky barriers *Φ*^e^_SB_ is significantly smaller than the *Φ*^h^_SB_, specifying that the Dirac-FeB_2_/MoS_2_ heterostructure possesses the n-type Schottky contact for all stacking configurations. Among those configurations, the FM-2 configuration exhibits the smallest *Φ*^e^_SB_, suggesting more efficient charge injection across the interface.

To gain deeper insights into the interfacial electronic coupling and the origin of the Schottky barriers, it is essential to analyze the projected density of states (PDOS) of the individual atoms in the Dirac-FeB_2_/MoS_2_ heterostructure. The PDOS provides valuable information on the orbital hybridization between the metallic FeB_2_ layer and the semiconducting MoS_2_ layer, as well as the alignment of the Fermi level relative to the band edges of MoS_2_. In particular, the interaction at the metal–semiconductor interface can induce metal-induced gap states (MIGS) within the band gap of MoS_2_, which play a crucial role in determining the Schottky barrier height and may lead to Fermi-level pinning (FLP). Therefore, a careful examination of the PDOS allows us to identify the degree of hybridization, the presence of MIGS, and the extent to which FLP influences the electronic properties of the Dirac-FeB_2_/MoS_2_ interface. The PDOS of the Dirac-FeB_2_/MoS_2_ heterostructure for all stacking configurations are shown in Fig. 4. It is evident that the electronic states crossing the Fermi level are mainly contributed by the metallic Dirac-FeB_2_ layer, while the MoS_2_ layer contributes negligible states near the Fermi level. Additionally, this finding confirms the absence of noticeable MIGS at the Dirac-FeB_2_/MoS_2_ interface, indicating that the electronic coupling between the two layers is weak and that the FLP is negligible.

**Fig. 5:**
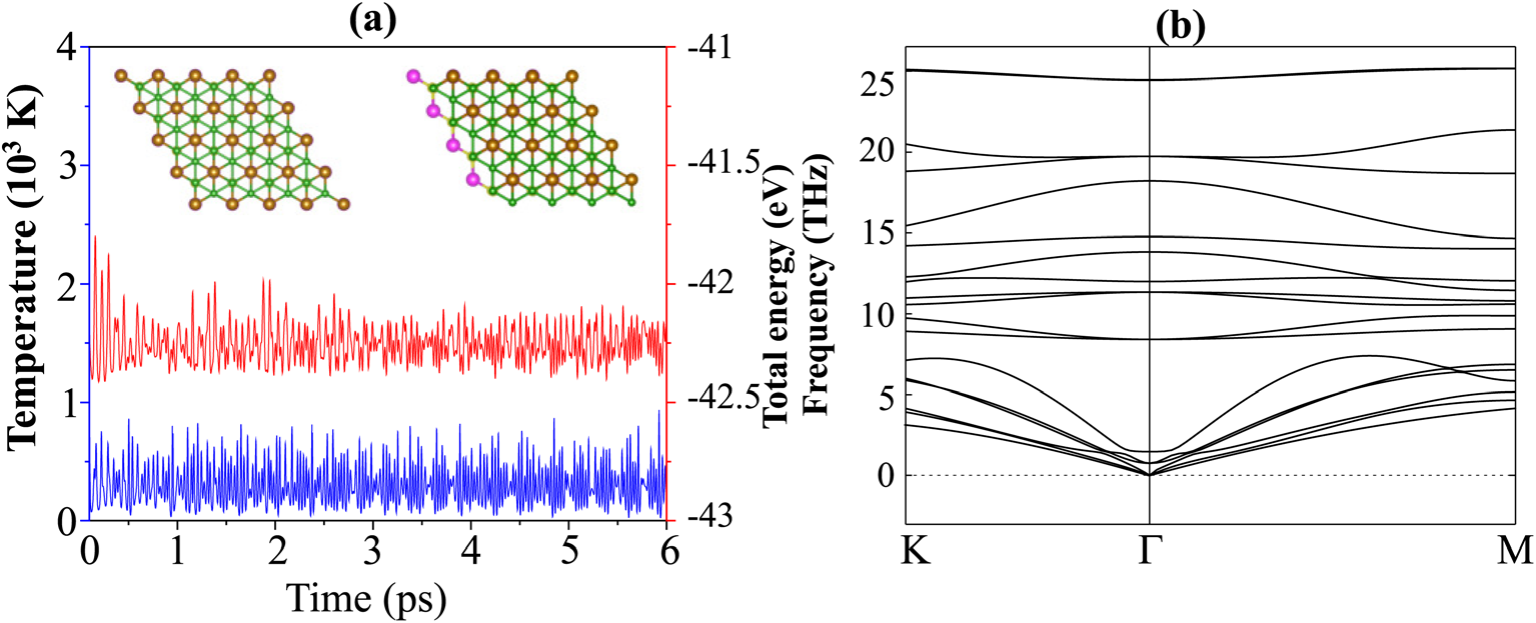
(a) The fluctuations of the temperature and total energy and (b) phonon spectra of the Dirac-FeB_2_/MoS_2_ heterostructure. The insets present the atomic structures of the Dirac-FeB_2_/MoS_2_ heterostructure before and after performing the AIMD simulation of 6 ps.

Furthermore, to examine the feasibility of experimentally synthesizing the Dirac-FeB_2_/MoS_2_ heterostructure, we evaluated its structural stability by performing mechanical response analysis, AIMD simulations, and phonon dispersion calculations, as illustrated in Fig. 5. The mechanical stability of the heterostructure can be examined by analyzing the in-plane elastic constants *C*_*ij*_ by fitting the strain–energy relationship within the small strain range (±1.5%). For a 2D hexagonal lattice, there are three relevant independent elastic constants, namely *C*_11_, *C*_12_, and *C*_66_ = [*C*_11_ − *C*_12_]/2. According to the Born–Huang stability criteria for a hexagonal system,^42^ the mechanical stability is guaranteed when: *C*_11_ > 0, *C*_66_ > 0 and *C*_11_ > |*C*_12_|. Our results show that the elastic constants *C*_11_, *C*_12_, and *C*_66_ are obtained to be 293.84, 113.46 and 90.19 N m^−1^, respectively. All these values satisfy the above criteria, confirming the mechanical robustness of the heterostructure. Additionally, Furthermore, the in-plane Young's modulus (*Y*) and Poisson's ratio (*ν*) are derived from the elastic constants as:3
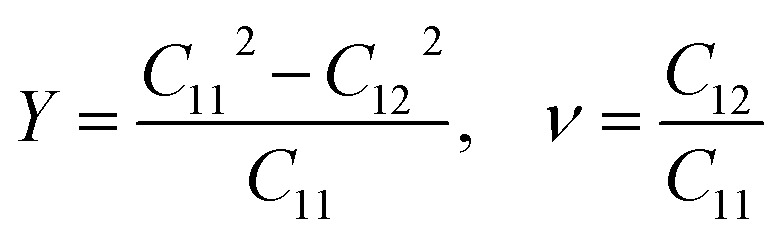
The Dirac-FeB_2_/MoS_2_ heterostructure exhibits a Young's modulus of *Y* = 250.02 N m^−1^ and a Poisson's ratio of *ν* = 0.39. The relatively large *Y* value indicates a strong in-plane stiffness and robust covalent bonding within the heterostructure, while the moderate Poisson's ratio suggests a reasonable degree of mechanical flexibility. These features confirm that the Dirac-FeB_2_/MoS_2_ heterostructure possesses excellent mechanical robustness.

To further confirm the thermal and dynamical stability of the Dirac-FeB_2_/MoS_2_ heterostructure, we performed *ab initio* molecular dynamics (AIMD) simulations at room temperature of 300 K for 6 ps with a time step of 1 fs. As shown in Fig. 5(a), both the temperature and total energy fluctuate slightly around their equilibrium values throughout the simulation, indicating the absence of any bond breaking or structural distortion during the thermal process. The insets present the atomic configurations of the heterostructure before and after the AIMD simulation, clearly demonstrating that the overall atomic framework remains intact. Moreover, the phonon dispersion spectrum of the Dirac-FeB_2_/MoS_2_ heterostructure, shown in Fig. 5(b), exhibits no imaginary frequencies across the Brillouin zone, thereby confirming its dynamical stability. The coexistence of thermal and vibrational stability strongly suggests that the Dirac-FeB_2_/MoS_2_ heterostructure is experimentally feasible and can maintain structural integrity under ambient conditions.

**Fig. 6:**
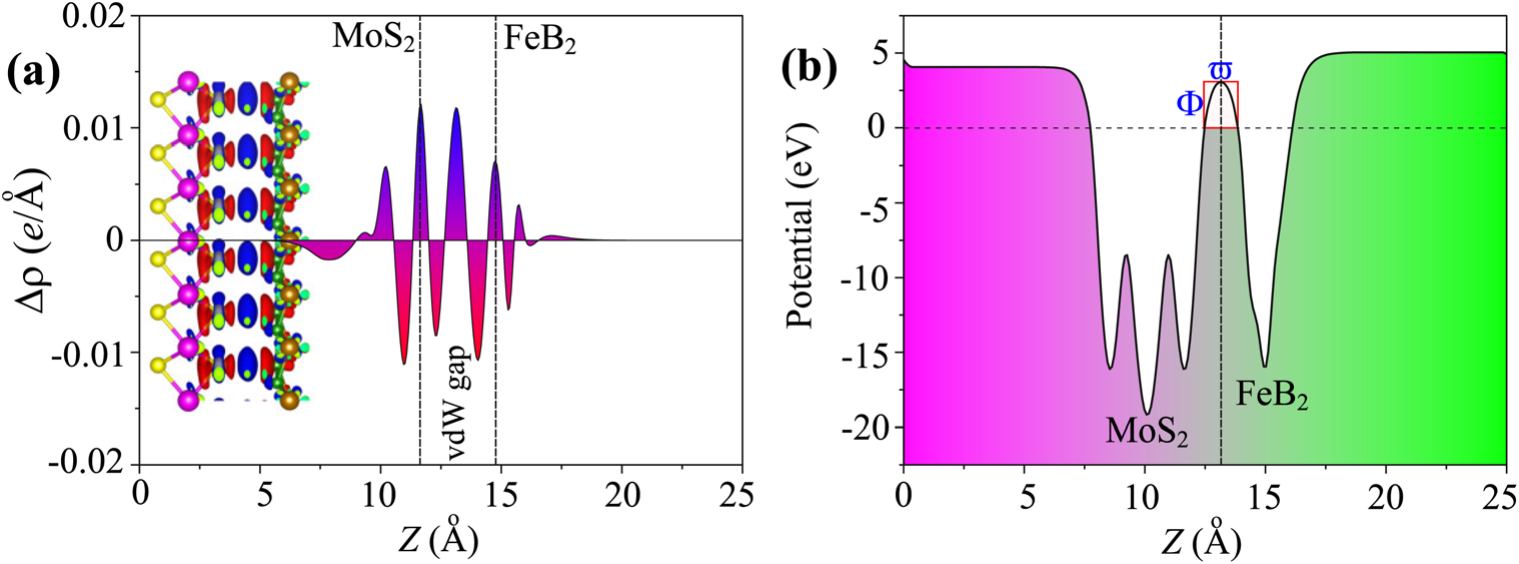
(a) Planar-averaged charge density difference and (b) electrostatic potential of the Dirac-FeB_2_/MoS_2_ heterostructure.

Fig. 6 summarizes the interfacial charge redistribution and the electrostatic potential profile of the Dirac-FeB_2_/MoS_2_ heterostructure. The charge density difference is defined as

4 Δ*ρ*(**r**) = *ρ*_FeB_2_/MoS_2__(**r**)*− ρ*_FeB_2__(**r**)*− ρ*_MoS_2__(**r**)

and its planar average along the direction normal to the interface is given by5
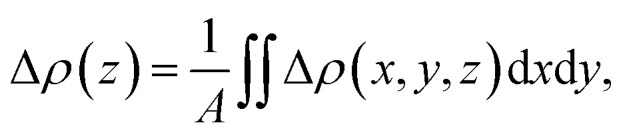


As shown in Fig. 6(a), the positive and negative values of Δ*ρ* correspond to charge accumulation and depletion regions, respectively. As shown in Fig. 6(a), the charge redistribution occurs mainly at the vdW gap between FeB_2_ and MoS_2_, indicating a weak but noticeable interfacial interaction. The electrons are transferred from the MoS_2_ layer to the FeB_2_ layer, forming an interfacial dipole directed from MoS_2_ to FeB_2_. To further elucidate the charge transfer mechanism, the planar-averaged electrostatic potential of the Dirac-FeB_2_/MoS_2_ heterostructure was calculated and is presented in Fig. 6(b). The potential difference (Δ*Φ*) between the two vacuum levels of FeB_2_ and MoS_2_ sides represents the built-in potential formed at the interface due to charge redistribution. This built-in electric field can facilitate charge separation and carrier transport across the interface, which is highly desirable for nanoelectronic and optoelectronic device applications.

To quantitatively evaluate the carrier tunneling across the vdW Dirac-FeB_2_/MoS_2_ heterostructure, the tunneling probability (*T*_P_) and tunneling specific resistivity (*ρ*_t_) are estimated using the semi-classical approximation:^43^
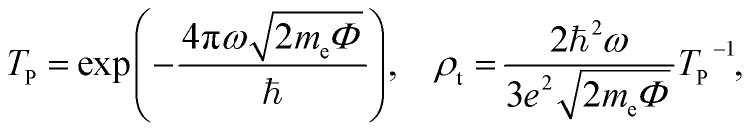
where *ω* and *Φ* represent the tunneling width and barrier height, respectively. Based on the electrostatic potential profile shown in Fig. 6(b), *ω* ≈ 1.37 Å and *Φ* ≈ 3.06 eV are obtained, yielding a tunneling probability of *T*_P_ ≈ 9% and a tunneling specific resistivity of *ρ*_t_ ≈ 1.82 × 10^−9^ Ω cm^2^. These results indicate a relatively high tunneling efficiency across the Dirac-FeB_2_/MoS_2_ interface. Notably, the obtained *ρ*_t_ value is comparable to those reported for both 3D and 2D (semi-)metal/semiconductor heterostructures,^44–47^ demonstrating that the Dirac-FeB_2_ layer can serve as an efficient electrode material for electronic device applications.

**Fig. 7:**
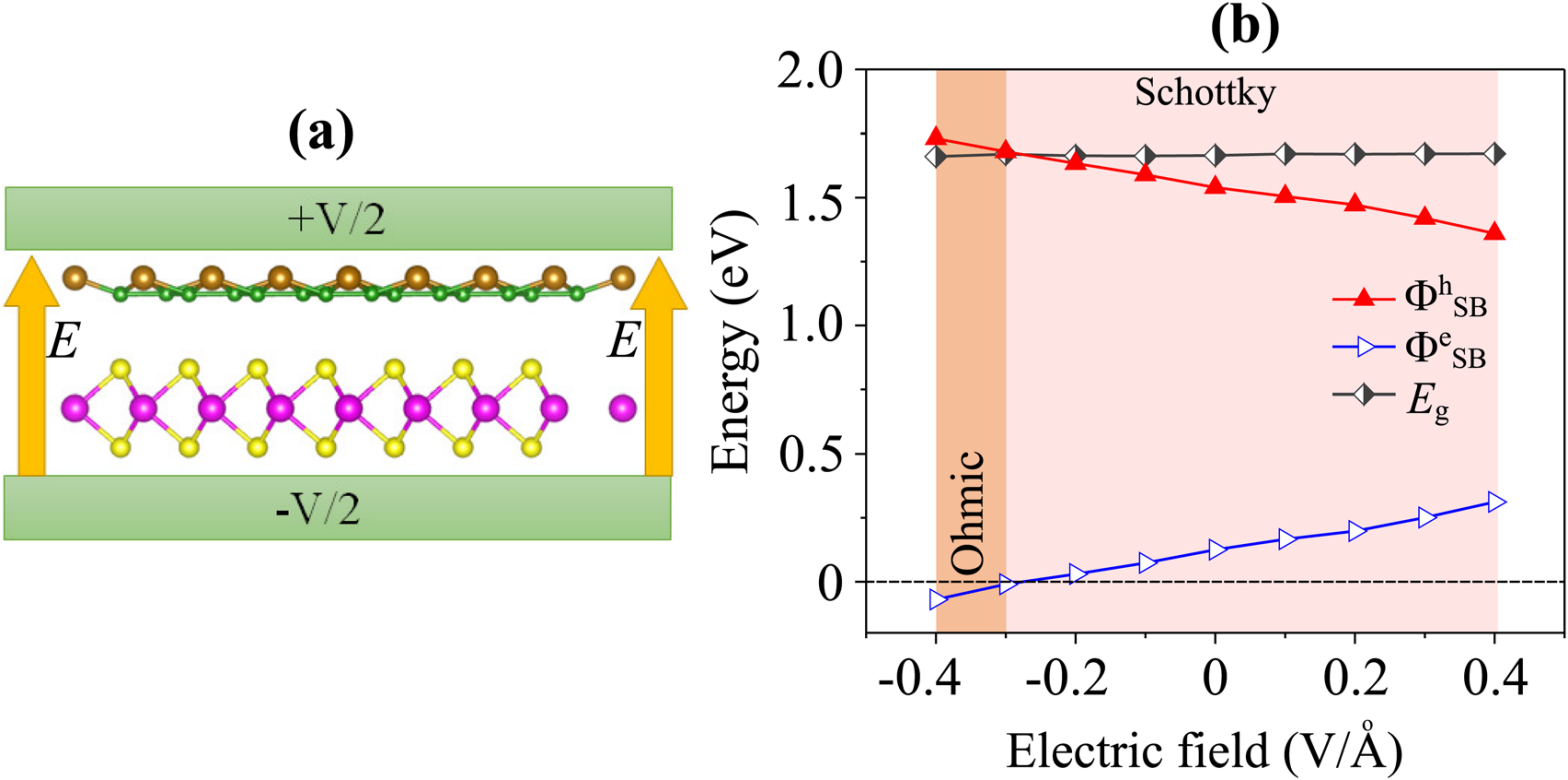
(a) Schematic model of applied electric field and (b) the variations in the Schottky barriers of the Dirac-FeB_2_/MoS_2_ heterostructure as a function of electric fields.

In practical electronic and optoelectronic devices, the external electric field plays a crucial role in tuning charge transport characteristics, contact resistance, and overall device performance. By modifying the potential landscape at the metal–semiconductor interface, an external field can effectively control carrier injection, Schottky barriers and contact types, thereby offering a powerful means for achieving high-efficiency and low-power device operation.^48–50^ To evaluate this effect, we investigated the electronic response of the Dirac-FeB_2_/MoS_2_ heterostructure under a perpendicular electric field, as illustrated in Fig. 7(a). The external electric field (*E*) is applied perpendicular to the heterostructure, with its direction defined as positive when pointing from the MoS_2_ layer toward the FeB_2_ layer. As shown in Fig. 7(b), the Schottky barrier for both electrons (*Φ*^e^_SB_) and holes (*Φ*^h^_SB_) exhibit a nearly linear dependence on the applied electric field. A negative *E* reduces *Φ*^e^_SB_ while slightly increasing *Φ*^h^_SB_, indicating enhanced electron injection from FeB_2_ into MoS_2_. When the field strength exceeds approximately −0.30 V Å^−1^, the *Φ*^e^_SB_ becomes negligible, leading to a transition from a Schottky to an ohmic contact. A similar behavior has been reported by Wang *et al.*,^48^ where an external electric field of about 0.3 V Å^−1^ induces a Schottky-to-ohmic transition in the graphene/GeC heterostructure. In contrast, a positive electric field increases the *Φ*^e^_SB_ and reduces the *Φ*^h^_SB_. In this case, *Φ*^e^_SB_ remains considerably smaller than *Φ*^h^_SB_, indicating that the Dirac-FeB_2_/MoS_2_ heterostructure retains its n-type Schottky contact character.

**Fig. 8:**
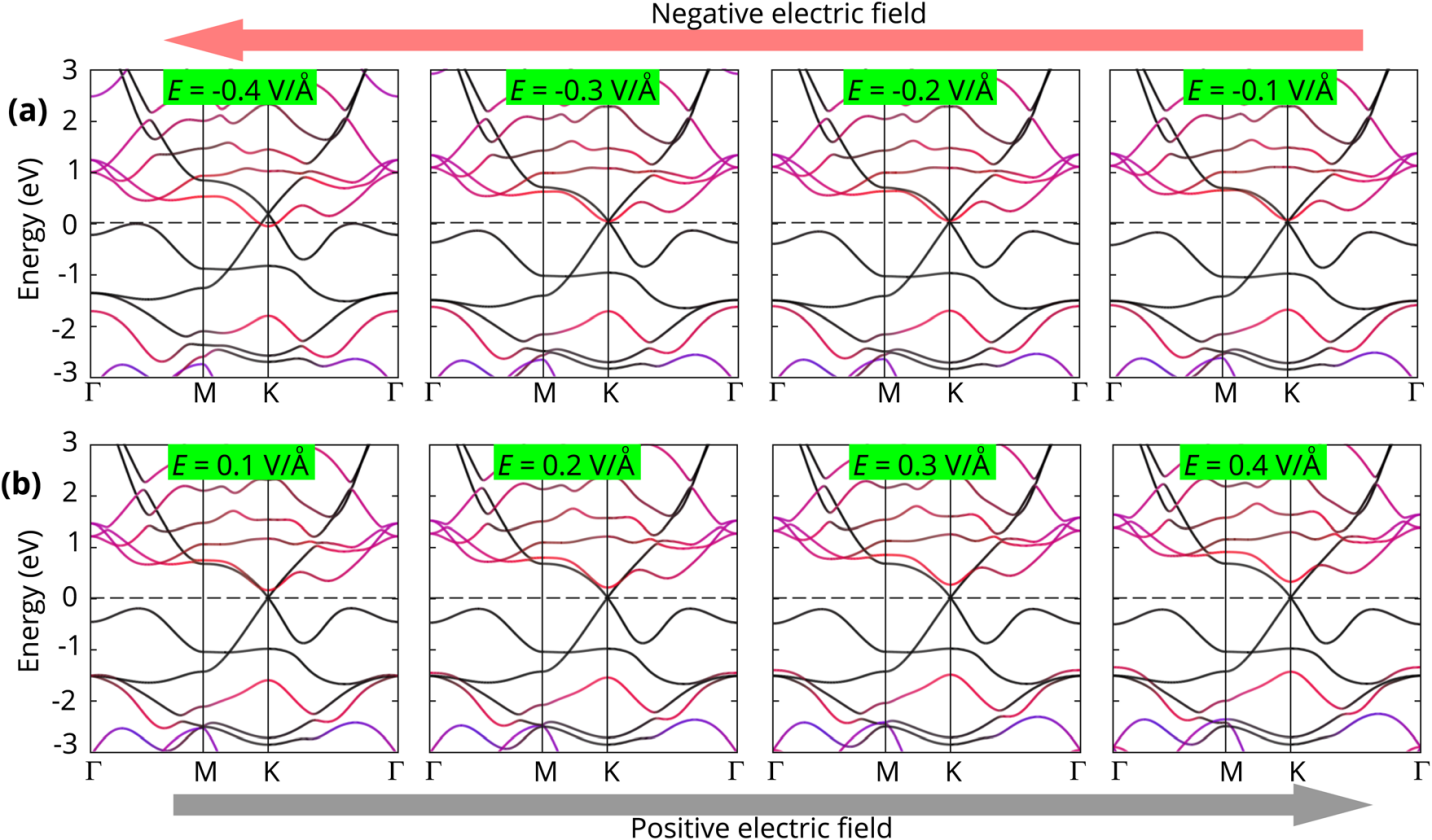
The projected band structures of the Dirac-FeB_2_/MoS_2_ heterostructure under (a) negative and (b) positive electric fields. The black and red lines represent the contributions of the Dirac-FeB_2_ and MoS_2_ layers, respectively.

To further elucidate the underlying mechanism of Schottky barrier modulation, we examined the projected band structures of the Dirac-FeB_2_/MoS_2_ heterostructure under different electric fields, as illustrated in Fig. 8. Under a negative electric field, the Dirac point of the FeB_2_ layer gradually shifts upward relative to the CBM of MoS_2_, resulting in a smaller electron Schottky barrier (*Φ*^e^_SB_). This shift facilitates charge transfer from FeB_2_ to MoS_2_, promoting efficient electron injection and eventually leading to an ohmic contact when *E* < −0.30 V Å^−1^. At the negative *E* = −0.4 V Å^−1^, the CBM of the MoS_2_ layer crosses the Fermi level of the Dirac-FeB_2_ layer, signifying the transformation from n-type Schottky contact to the n-type ohmic contact. In contrast, under a positive electric field, the Dirac point of FeB_2_ moves downward, increasing *Φ*^e^_SB_ and decreasing the hole barrier *Φ*^h^_SB_. The opposite band bending in this regime suppresses electron tunneling and weakens the interfacial charge transfer. Despite this modulation, the Fermi level remains closer to the MoS_2_ CBM than its VBM, confirming that the Dirac-FeB_2_/MoS_2_ heterostructure consistently retains its n-type Schottky contact nature across the entire field range. All these findings demonstrate that the Schottky barriers and contact types in the Dirac-FeB_2_/MoS_2_ heterostructure can be effectively tuned by an external electric field. The field-induced modulation enables a reversible transition between Schottky and ohmic contacts, allowing precise control over carrier injection and transport characteristics at the interface. Such tunability provides a promising strategy for designing next-generation field-effect transistors, Schottky diodes, and other nanoelectronic or optoelectronic devices based on the Dirac-FeB_2_/MoS_2_ heterostructures with high efficiency and low contact resistance.

**Fig. 9:**
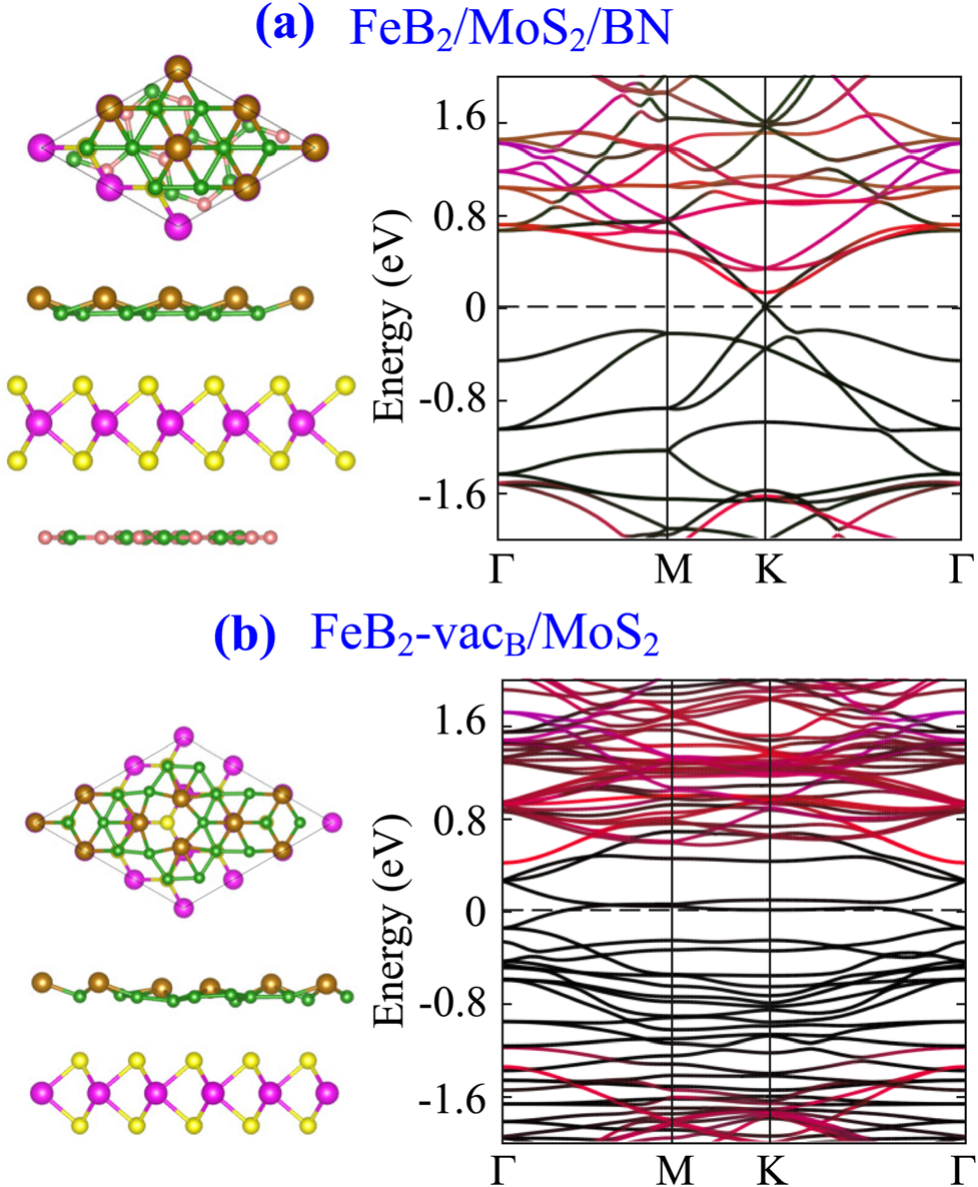
Atomic structures and band structures of FeB_2_/MoS_2_ heterostructure with the presence of (a) BN substrate and (b) B vacancy (vac_B_).

In practical device configurations, metal–semiconductor heterostructures are typically supported on a substrate and may also contain intrinsic defects at the interface. Therefore, to assess the robustness of the contact behavior, we further examine the influence of a representative hexagonal BN substrate as well as vacancy defects on the FeB_2_/MoS_2_ heterostructure. The atomic structures and band structures of these configurations are depicted in Fig. 9. Our results show that the presence of a BN substrate gives rise to a slight reduction in *Φ*^e^_SB_ to 0.120 eV, while the presence of a B vacancy defect leads to an increase in *Φ*^e^_SB_ to 0.382 eV. These findings indicate that the contact characteristics are relatively robust against substrate effects, whereas defects can significantly modify the Schottky barrier through the introduction of localized states and altered charge transfer at the interface.

## 4 Conclusions

In this work, we have explored the structural, electronic, and interfacial properties of the Dirac-FeB_2_/MoS_2_ vdW heterostructure using first-principles calculations. The FeB_2_ monolayer exhibits a stable planar hexacoordinate structure with a Dirac-like metallic nature, which is well preserved upon stacking with semiconducting MoS_2_. The Dirac-FeB_2_/MoS_2_ heterostructure is energetically, mechanically, thermally and dynamically stablie and forms an n-type Schottky contact with a small electron barrier height and high tunneling probability, facilitating efficient carrier injection across the interface. Furthermore, the external electric field provides an efficient way to modulate the Schottky barrier and contact type. A negative field reduces the electron barrier and induces an ohmic contact, whereas a positive field increases it while preserving the n-type character. These results highlight the Dirac-FeB_2_/MoS_2_ heterostructure as a promising candidate for next-generation tunable, low-resistance, and high-efficiency 2D electronic and optoelectronic devices.

## Conflicts of interest

There are no conflicts to declare.

## Data Availability

The data that support the findings of this study are available from the corresponding author upon reasonable request.
